# Pupil function design for multifocal confocal, STED, and isoSTED microscopy

**DOI:** 10.1364/AO.416585

**Published:** 2021-06-16

**Authors:** Dong-Ryoung Lee, Joerg Bewersdorf

**Affiliations:** 1 Department of Cell Biology, Yale School of Medicine, 333 Cedar Street, New Haven, Connecticut 06520, USA; 2 Department of Biomedical Engineering, Yale University, 333 Cedar Street, New Haven, Connecticut 06520, USA

## Abstract

Point scanning super-resolution microscopy techniques such as stimulated emission depletion (STED) microscopy are powerful tools to observe biological samples at sub-diffraction limited resolution in three dimensions. However, scanning the sample with only a single beam limits the imaging speed in these microscopes. Here, we propose a concept to increase this speed by introducing highly flexible multifocal illumination and detection. We introduce phase patterns in the objectives’ pupil planes to create arrays of foci in the sample plane with negligible loss of laser power. High uniformity of these foci’s intensities is achieved by iteratively applying a weighted Gerchberg–Saxton phase retrieval algorithm. We characterize the performance of this iterative approach numerically and present simulation results that demonstrate the high quality of the focus arrays for future implementations in laser-scanning STED and isoSTED microscopes. The same approach can also be applied in diffraction-limited confocal laser scanning microscopy.

## 1. INTRODUCTION

Optical microscopy has been an essential tool in biology for centuries. Over the past few decades, confocal laser scanning microscopes with their ability to reject out-of-focus background light have become indispensable to observe biological samples in three dimensions. However, laser scanning microscopes read out signal only from the focal region, requiring to move the focus across the sample to create an image. This approach leads to compromises among the obtained resolution, field of view (FOV), and imaging speed. To address this compromise, multifocal imaging methods using, for example, a spinning disk [1] or a digital micromirror device [2] have been introduced.

In parallel, super-resolution microscopy techniques have been developed to overcome the limit of diffraction of conventional far-field optical microscopy and visualize cell morphology and molecular dynamics at the nanoscale. These new imaging techniques include stimulated emission depletion (STED) microscopy [3] as well as stochastic single-molecule switching techniques [4].

STED microscopy is a laser-scanning microscopy technique that, in addition to an excitation laser, uses a separate laser beam to prevent fluorescence emission through STED. To quench fluorescence only in the periphery of the excitation focus, STED microscopes shape the focal spot of the depletion beam to have zero intensity at its center. This is usually achieved by phase masks in a conjugate plane of the objective’s back pupil. Through this approach, STED microscopes reduce the size of the effective focal spot, as defined by the area from which fluorescence is emitted, by typically a factor of five or more in two or three dimensions compared to the diffraction-limited focus [5,6]. Due to the elongated shape of the diffraction-limited focus, a STED microscope’s axial resolution is typically at least 2.5 times worse than its lateral resolution. To overcome this limitation, isoSTED microscopy was developed [7,8], which, by using coherent illumination through two opposing objectives in a 4Pi geometry [9–11], creates an axially sharpened focus. isoSTED instruments have demonstrated 20–50 nm isotropic resolution in all dimensions [7,12,13].

The strongly reduced effective focal volume—in the case of isoSTED, about three orders of magnitude compared to a diffraction-limited focus—comes, however, at the cost of imaging speed since the sample needs to be scanned at much smaller pixel sizes to meet Nyquist sampling conditions. Especially for three-dimensional (3D) image stacks, which require the acquisition of multiple two-dimensional (2D) images, scanning a large FOV with a single excitation focus is a severe challenge. To overcome this limitation, there have been several developments of multifocal STED microscopes. One approach [14] created four pairs of excitation and depletion beams using Wollaston prisms; however, the positions of the focal spots were fixed, as they were governed by the separation angles introduced by the used Wollaston prisms. Alternatively, a grid depletion pattern introduced by a grating combined with widefield excitation and camera detection has been applied to parallelize STED and (REversible Saturable OpticaL Fluorescence Transitions) RESOLFT microscopes [15–19]; however, the high level of parallelization (up to 100,000) requires lasers of low repetition rate and high pulse energy, which are not standard for STED microscopy, and the use of cameras limits the imaging speed and the choice of FOV.

Another approach used a phase mask in the pupil plane of the objective to engineer focal spots using a direct search algorithm [20]. This approach is particularly attractive because of the flexibility in possible focus pattern designs provided by the used spatial light modulator (SLM) and its compatibility with SLM-based aberration correction [21].

While many of these approaches have demonstrated impressive 2D STED images, none has demonstrated the application to 3D super-resolution STED microscopy yet, where depletion patterns deplete the excitation focal volumes in all three spatial directions, including the axial direction. This extension is challenging due to the required 3D interference patterns, which are less robust to small deviations in the wavefronts [21,22].

We set out to design an SLM-compatible approach for parallelized 3D STED microscopy, in particular for future integration into a parallelized isoSTED instrument. Instead of the previously demonstrated direct search algorithm [20], which showed a fairly low diffraction efficiency (68% for 10×10 focal spots), we chose a Gerchberg–Saxton (GS) algorithm, which has demonstrated higher diffraction efficiencies (94% for 10×10 focal spots) in parallelized optical tweezers [23]. This is of particular importance in STED microscopy since high intensities are required in each depletion focus, and the available laser power can easily reach its upper limit in parallelized setups. In addition, in the same publication, a weighted GS (WGS) algorithm was introduced to produce foci of nearly equal intensities [23]. Since STED microscopy also requires high uniformity among all foci for uniform resolution over the whole FOV, we iteratively apply a similar GS algorithm with weight factors.

Here, we apply a WGS algorithm to parallelize STED and isoSTED microscopes using custom phase masks in the conjugate planes of the objectives’ back pupils. We describe using a phase retrieval algorithm [24,25] iteratively to guide the design of the phase patterns. Our approach results in nearly equal intensities among all foci while maintaining high diffraction efficiency (>80%; defined as the sum of laser powers concentrated in the desired spots divided by the total incident laser power). To the best of our knowledge, this is the first work that computationally shows multifocal STED with equal intensity and high efficiency as well as excellent doughnut minima quality for all focal spots. Additionally, we present simulations of its application in multifocal isoSTED microscopy using the proposed method and compare them to single-focus isoSTED microscopy.

## 2. ALGORITHM DESCRIPTION

**Fig. 1.**
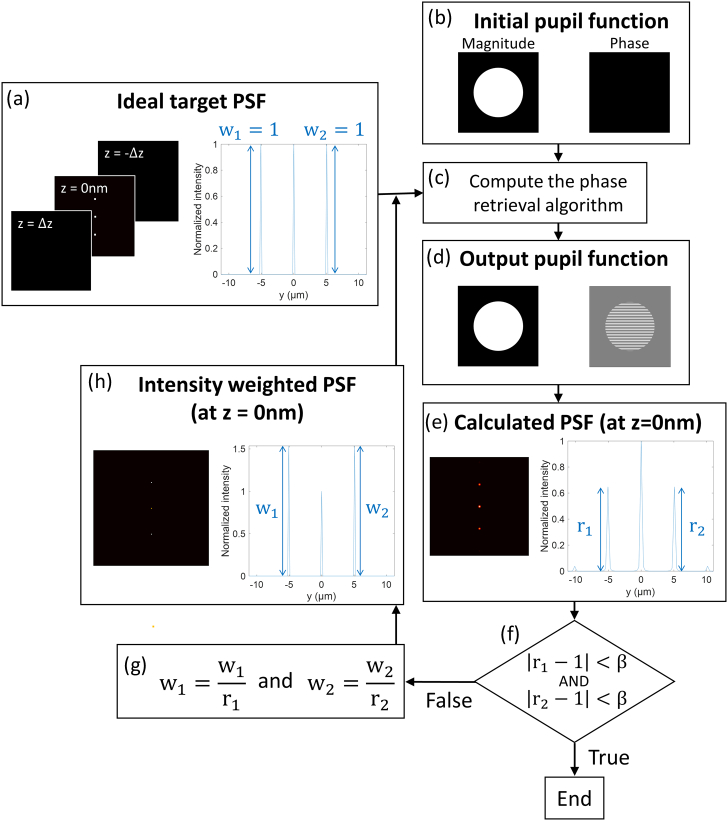
Diagram for iterative phase retrieval algorithm for the generation of phase grating profiles for a specific spot distribution. $r_{1}$, $r_{2}$, intensity ratios; $w_{1}$, $w_{2}$, weight factors.

Arrays of foci are generated by laser light diffraction at the custom grating patterns displayed on the phase-only masks. To generate these patterns, we define our desired 3D intensity distribution in the sample space [real space; Fig. 1(a)] and estimate the phase component of the corresponding pupil function of the objective, i.e., the Fourier transform of the intensity distribution in the focal plane, using a modified GS phase retrieval algorithm [24–26] [Fig. 1(d)]. We calculate the intensity distribution near the focal plane from the estimated pupil function following the theory by Richards and Wolf [27,28] as

(1) $$h(\vec{r})=C_{1}{\left|\vec{E}(\vec{r})\right|}^{2},$$

where $C_{1}$ is a constant. $\vec{E}(r,\phi ,z)$ is the electric field vector describing the incident light expressed in cylindrical coordinates and is calculated as

(2) $$\begin{array}{ll} \vec{E}(r,\phi ,z) & =iC_{2}\iint_{\Omega }\sin\theta \cdot \sqrt{\cos\theta }\cdot A(\theta ,\phi \prime )\cdot \vec{P}(\theta ,\phi \prime )\\ & \;\cdot e^{i\{\frac{2\pi n}{\lambda }[z\cos\theta +r\sin\theta \cos(\phi \prime -\phi )]+\Delta \alpha (\theta ,\phi \prime )\}}d\theta d\phi \prime , \end{array}$$

where $C_{2}$ is a constant, Ω is the solid angle representing the objective aperture, A(θ,ϕ) is the amplitude function of the input light, $\vec{P}(\theta ,\phi )$ is the polarization function in the image field, n is the refractive index, λ is the wavelength, and Δα(θ,ϕ) is the phase delay introduced by the phase mask.

The polarization function in Eq. (2) is converted from the polarization vector of input light by multiplying the conversion matrix from the object field to the image field [28] and expressed as

(3) $$\begin{array}{ll} \vec{P}(\theta ,\phi ) & =\left[\begin{array}{ccc} 1+(\cos\theta -1)\cos^{2}\phi & (\cos\theta -1)\cos\phi \sin\phi & -\sin\theta \cos\phi \\ (\cos\theta -1)\cos\phi \sin\phi & 1+(\cos\theta -1)\sin^{2}\phi & -\sin\theta \sin\phi \\ \sin\theta \cos\phi & \sin\theta \sin\phi & \cos\theta \end{array}\right]\\ & \;\times \left[\begin{array}{c} P_{x}\\ P_{y}\\ P_{z} \end{array}\right]. \end{array}$$

**Fig. 2.**
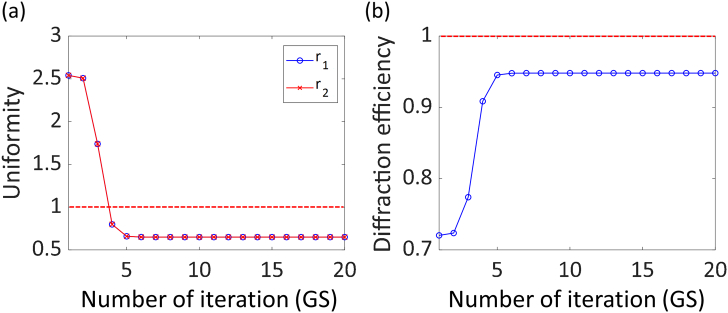
Uniformity and diffraction efficiency for Gerchberg–Saxton algorithm at each iteration. (a) Relative intensities between the peaks and (b) diffraction efficiency.

The pupil function generated by using the GS algorithm forms focal spots at the desired locations, but their intensities are usually non-uniform [Fig. 1(e)] because this algorithm aims at maximizing the diffraction efficiency, having no bias towards uniformity [23]. This can be seen in Fig. 2, which shows the quick convergence of the GS algorithm to a maximum diffraction efficiency of more than 90% after only four iterations while also revealing the failure to produce foci of uniform intensity. This uniformity between peaks is measured by calculating the ratios of the peak intensities (defined as the maxima in the regions surrounding each focal spot) of the different peaks to an arbitrarily selected peak (here the center peak), $r_{i}$ (here $r_{1}$ and $r_{2}$) [Fig. 1(e)] in order to compare the peak intensities directly among foci. (This approach is similar to the one described by Di Leonardo *et al.* [23], which minimizes the differences between the maximum and minimum intensities.) To solve this problem and optimize the phase patterns for best intensity uniformity among the focal spots, we developed a strategy where we repeat the GS phase retrieval algorithm with adjusted target intensity distributions until it meets preset uniformity criteria: when both $r_{1}$ and $r_{2}$ come within a range of a parameter β to unity, the iterative loop is terminated [Fig. 1(f)]. Otherwise, we modify the ideal intensity distribution [Fig. 1(a)] by multiplying the respective peaks with weight factors, $w_{i}$, which are the inverse of $r_{i}$ [Fig. 1(h)], so that the ratios become one and rerun the phase retrieval algorithm.

This approach is similar to an approach described by Waller *et al.* [29], in which the authors introduced a modified GS algorithm for a parallelized optical tweezers system that iteratively modifies the magnitude and the phase of the pupil function with weighting terms on the amplitudes of the individual foci to achieve high uniformity between them. In contrast to Waller *et al.*, we show here that variations in phase patterns alone are sufficient to realize multiple foci of equal intensity.

**Fig. 3.**
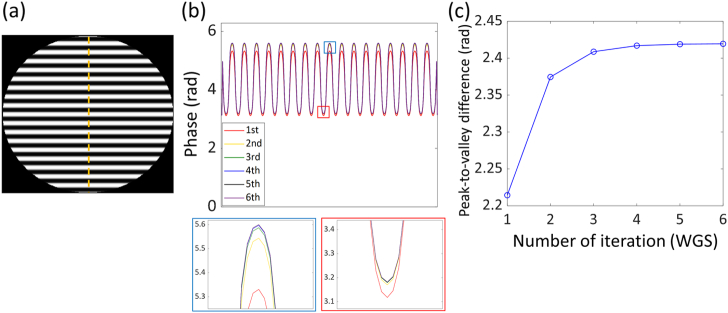
(a) Grating pattern of the pupil function for three spots in the sample plane; (b), (c) cross section and peak to valley difference of the grating pattern at each iteration of the weighted Gerchberg–Saxton algorithm. Insets show the peak and the valley as indicated by the blue and red boxes in (b), respectively.

We refer to our modified GS algorithm as a WGS algorithm. During the iterative procedure of WGS, the amplitude of the grating pattern is modulated as shown in Fig. 3. Figure 3(a) shows the example of the pupil function for three spots, and Figs. 3(b) and 3(c) show the cross section along the center line, indicated by the dashed line in Fig. 3(a), and the amplitude of the grating pattern at each iteration, respectively.

## 3. SIMULATION RESULTS

### A. Multiple Focal Spots

**Fig. 4.**
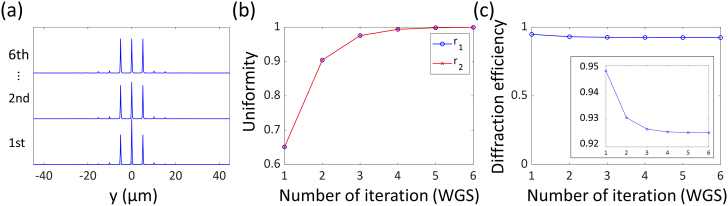
(a) Intensity distribution at each iteration of the WGS algorithm, (b) relative intensities between the peaks, and (c) diffraction efficiency. Inset shows the same plot as (c) with a rescaled y axis.

We tested our approach first for a 2D application with simulations choosing a wavelength of 775 nm, numerical aperture of 1.35, refractive index of 1.406, and circular polarization to match typical experimental parameters. We generated an 8705×8705×3 matrix with a pixel size of $d_{\text{xy}}=10\;nm$ and $d_{z}=775\;nm$. To minimize numerical errors, we chose a small pixel size in the lateral plane and a large FOV, being limited only by the available computer memory. The pixel size in the z direction was deliberately chosen to be larger than the diffraction limit to suppress intensities outside the focal plane but not pose unnecessary constraints onto the focal shape within the diffraction-limited range. For a pattern of three focal spots 5 µm apart along the y axis, we set up the matrix to be zero except at positions (4353, 3853, 2), (4353, 4353, 2), and (4353, 4853, 2) where it was one. Figure 4(a) shows the intensity distributions as $r_{1}$ and $r_{2}$ [Fig. 4(b)] approach one over the iterative optimization process. With β=0.001, the loop was terminated after six iterations with $r_{1}=r_{2}\approx 0.9996$. Besides uniform focal intensities, a high diffraction efficiency is an important design factor since the available laser power in STED microscopes is often limited and background and bleaching outside the focal regions should be minimized [30]. Figure 4(c) shows the diffraction efficiency at each iteration. As the amplitude of the phase grating is modulated, the diffraction efficiency drops slightly by 2.2%, resulting in a still very high diffraction efficiency of 92.6% after the last iteration.

**Fig. 5.**
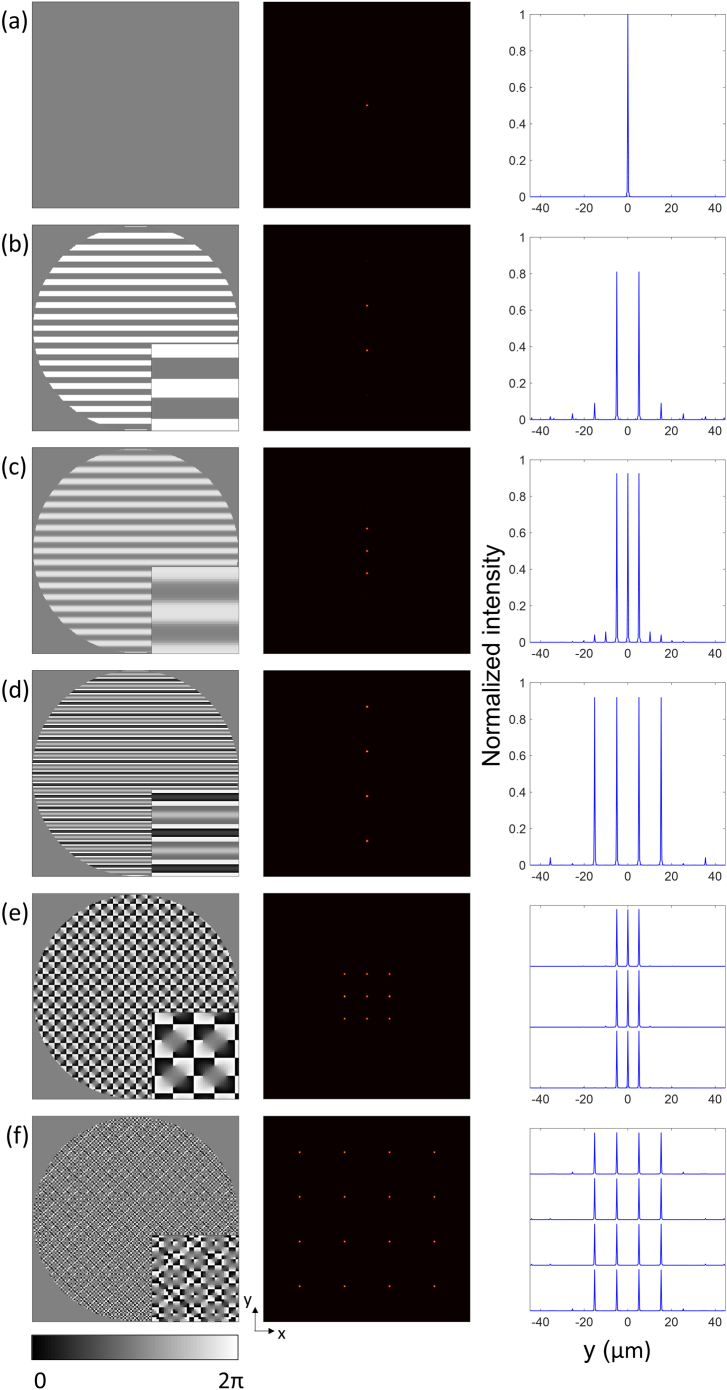
Optimized pupil planes, and corresponding simulation results of intensity distributions at the focal plane and their intensity profiles. The amplitudes of the line profiles are divided by the number of foci, resulting in a perfect diffraction efficiency represented by the value one. The stacked line graphs in (e) and (f) show the profiles of each row of foci. Offsets between profiles are one. Insets show zoomed-in areas of the pupil plane patterns. Scale bars, 5 µm.

Depending on the application type, the number of focal spots and the pattern of the spot array can be easily adjusted. For example, when the beam scanner is composed of a resonant mirror for the fast axis and a galvanometer mirror for the slow axis, multiple spots along the slow axis can improve the speed of raster scanning because fewer lines need to be scanned by the slower galvanometer mirror. In other words, n-fold parallelized foci provide n-fold higher imaging speed for a given sample area without compromising spatial resolution or sampling. We tested our approach numerically with different patterns of focal arrays and analyzed two key features of the intensity distribution: their intensity uniformity and diffraction efficiency. Figure 5 shows the optimized phase patterns and the corresponding simulated intensity distributions in the focal plane. The intensity distributions in the focal plane are normalized to 1/(number of focal spots). The total intensity of the foci is therefore a direct measure of the diffraction efficiency for each pattern. The iteration termination criterion β was set to 0.001 for all cases, which means that all intensity uniformities are better than 99.9%. The diffraction efficiencies in the simulation results are 81.0%, 92.6%, 91.9%, 93.3%, 90.6% for 2×1,3×1,4×1,3×3, and 4×4 spots, respectively. All phase patterns are 315×315 pixels large in our simulations, which is nearly identical in size to the phase masks used with liquid crystal SLMs in experimental STED setups [8].

### B. Multifocal Single-Objective STED Microscopy

**Fig. 6.**
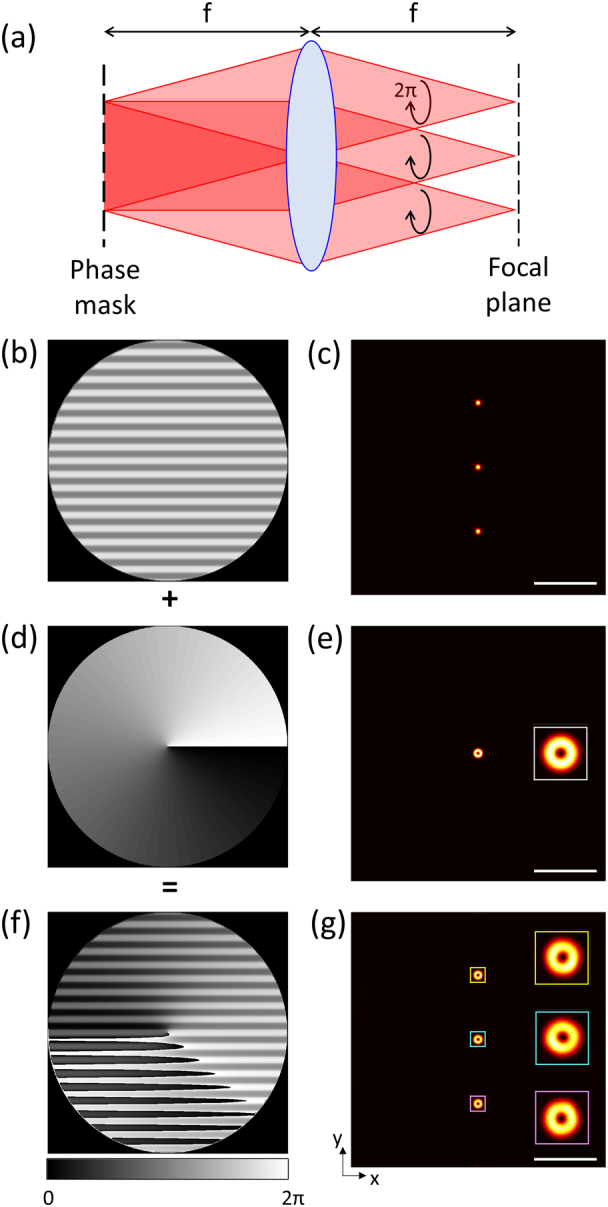
(a) Geometry of the beam path to generate the multiple doughnut-shaped beams; (b), (d), (f) grating, vortex, and sum patterns and (c), (e), (g) corresponding intensity distributions, respectively. Scale bars, 5 µm.

Figure 6(a) shows the geometry of a beam path to generate multiple doughnut-shaped beams for STED microscopy and related approaches such as RESOLFT microscopy [31]. The phase grating designed by the iterative phase retrieval algorithm splits beams into multiple orders [Figs. 6(b) and 6(c)]. A vortex phase pattern is used to create a doughnut-shaped focal spot [Figs. 6(d) and 6(e)] [28]. By summing the two phase patterns, multiple doughnut-shaped focal spots are created [Figs. 6(f) and 6(g)] that can be used for the depletion beam.

**Fig. 7.**
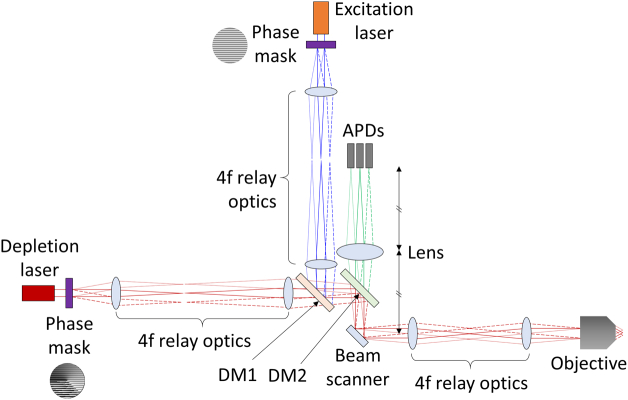
Possible system schematic of a multifocal (single-objective) STED setup. DM1, DM2, dichroic mirrors; APDs, avalanche photo diodes. To simplify the figure, transmissive phase masks are used, but they can be replaced with reflective SLMs.

For future implementations in a single-objective geometry, we suggest a beam path similar to the one presented in [20]. We propose to use a liquid crystal SLM as a phase mask for both smoothly and sharply varying phase patterns, rather than using microfabricated dielectric phase masks or deformable mirrors. Phase masks are placed in the conjugate planes of the objective’s back pupil in the excitation and depletion beam paths, as shown in Fig. 7. The masks are designed to create arrays of excitation foci as well as doughnut-shaped depletion foci. Lens systems in 4f-configuration image these phase patterns onto the scanning mirror (used for beam scanning) and then into the back pupil plane of the objective to create multiple laser foci. Fluorescence emitted from these foci is then collected by the objective and relayed to multiple avalanche photodiodes (or similar sensitive detectors), each detecting the fluorescence stemming from one of the excitation foci in a confocal detection geometry.

**Fig. 8.**
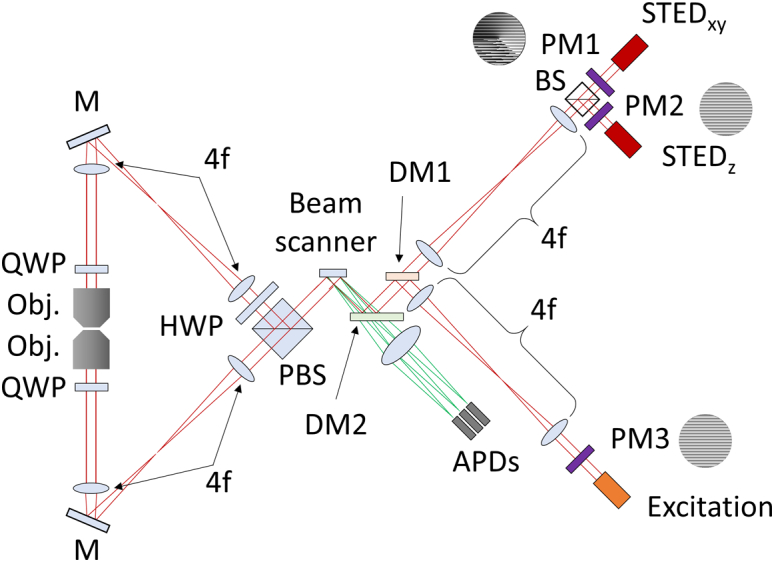
Possible system schematic of a multifocal isoSTED setup. PM1, PM2, PM3, phase masks; BS, beam splitter; DM1, DM2, dichroic mirrors; PBS, polarizing beam splitter; HWP, half-wave plate; QWP, quarter-wave plate; Obj., objective lens; APDs, avalanche photo diodes.

### C. Multifocal isoSTED Microscopy

We next simulated our approach in an isoSTED microscope setting to realize parallelized STED microscopy with isotropic 3D super-resolution. Figure 8 shows the system schematic to implement multifocal isoSTED microscopy. Two depletion patterns featuring a common focal zero are required [13]: one is the sum of the grating and the vortex patterns (PM1) for lateral depletion (${STED}_{\text{xy}}$), and the other one is the grating pattern alone (PM2), utilizing interference of the two counterpropagating beams passing through the two opposing objectives for axial depletion (${STED}_{z}$). After the two different depletion beams are combined, beams propagate to the beam scanner as described earlier, and are then split by a polarizing beam splitter cube [7] and directed to the back-pupil planes of each objective lens. Identical multiple spots are focused into the same focal plane by both objectives and interfere with each other. The excitation beam propagates through the optics in a corresponding manner. Emitted fluorescence travels back through both objectives, and is combined by the polarizing beam splitter cube and detected in the same way as in the parallelized single-objective STED setup described above.

The 3D intensity distribution of Eq. (1) can be extended to isoSTED systems by the following equation [16]:

(4) $$h_{4\;Pi}(x,y,z)={\left|\vec{E}(x,y,z)+\left[\begin{array}{ccc} 1 & 0 & 0\\ 0 & 1 & 0\\ 0 & 0 & -1 \end{array}\right]\vec{E}(x,y,-z)e^{i\varphi }\right|}^{2},$$

where φ is the phase delay between the two fields (focused by the two objective lenses) in the focal plane.

The depletion beam quenches excited fluorophores where they are overlapped. When the excitation foci are confined by the depletion beams, fluorescence emission is inhibited everywhere but at the centers of the foci. The effective focal spots of the isoSTED microscope can be calculated as described before [32] as

(5) $$h_{\text{eff}}(\vec{r})=h_{\text{exc}}(\vec{r})\exp(-\ln(2)h_{4\;Pi,STED}(\vec{r})/I_{s}),$$

where $I_{s}$ is the effective saturation intensity at which the probability of fluorescence emission is reduced by half, and $h_{\text{exc}}$ is the excitation intensity distribution.

**Fig. 9.**
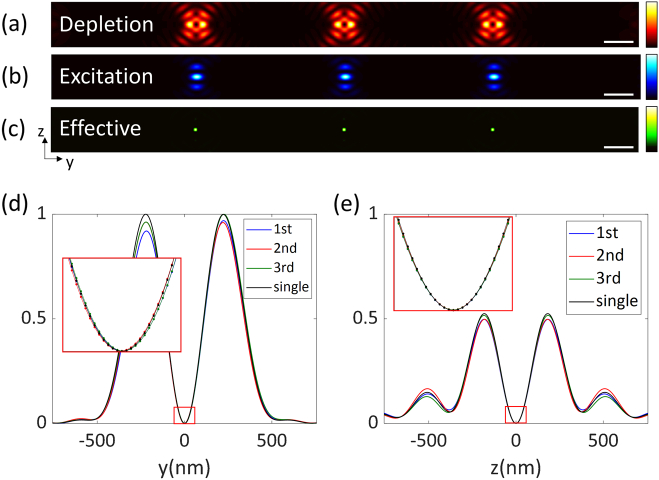
(a), (b), (c) Simulation results for depletion, excitation, and effective focal spots and (d), (e) intensity profiles of a multifocal isoSTED microscopy and a conventional single-focal isoSTED microscopy around the center of each focus along y and z axes, respectively. Scale bars, 1 µm.

Figures 9(a)–9(c) show the simulation results of depletion, excitation, and effective focus patterns in the yz plane, respectively. Figures 9(d) and 9(e) show the intensity profiles of the three different foci of the multifocal isoSTED layout and the focus of single-focal isoSTED microscopy around the center of each depletion focus along the y and z axes, respectively, to compare the steepness and minimum values that determine the resolution. When the laser intensity is adjusted so that the maximum intensity of $h_{4\;Pi,STED}$ in Eq. (5) is the same for both the multifocal and single-focal cases, the intensity profiles are equally steep and, importantly, both have zeros at their centers. In addition, to remove the side lobes of the effective foci above and below the focal plane, we applied defocus to the ${STED}_{z}$ pattern [33].

## 4. CONCLUSION

In conclusion, we propose WGS, an algorithm that iteratively executes a GS algorithm with target intensity distributions being adjusted between iterations to optimize the phase pattern at the pupil plane. We demonstrated its excellent performance by simulating results for parallelized 2D STED and 3D isoSTED microscopy. Our results show that our approach can generate versatile patterns of foci arrays with high intensity uniformity among the foci and diffraction efficiency. Importantly, high-quality interference minima can be obtained in each STED focus even in the case of the complex 3D depletion foci of isoSTED microscopy. These findings expand on previously reported algorithms that have introduced similar phase masks for optical tweezers [23,29] and 2D super-resolution STED microscopy [20]. Consequently, our method is equally applicable to parallelizing other laser-scanning microscopy approaches such as confocal microscopy and RESOLFT microscopy. Therefore, the proposed method can be useful for a large range of applications to improve imaging speed.

## Data Availability

Data underlying the results presented in this paper are not publicly available at this time but may be obtained from the authors upon reasonable request.
